# Therapeutic Potential of Rituximab in Managing Hepatitis C-Associated Cryoglobulinemic Vasculitis: A Systematic Review

**DOI:** 10.3390/jcm12216806

**Published:** 2023-10-27

**Authors:** Andreea Covic, Irina Draga Caruntu, Alexandru Burlacu, Simona Eliza Giusca, Adrian Covic, Anca Elena Stefan, Crischentian Brinza, Gener Ismail

**Affiliations:** 1 Nephrology Department, Dialysis and Renal Transplant Center, “Dr. C.I. Parhon” University Hospital, 700503 Iasi, Romania; andreea.covic@gmail.com (A.C.); adrian.covic@umfiasi.ro (A.C.); anca0729@gmail.com (A.E.S.); 2 Faculty of Medicine, ‘Grigore T. Popa’ University of Medicine, 700115 Iasi, Romania; irina.caruntu@umfiasi.ro (I.D.C.); alexandru.burlacu@umfiasi.ro (A.B.); crischentian.brinza@d.umfiasi.ro (C.B.); 3 Department of Morpho-Functional Sciences I—Histology, Pathology, “Grigore T. Popa” University of Medicine and Pharmacy, 700115 Iasi, Romania; 4 Department of Interventional Cardiology, Cardiovascular Diseases Institute “Prof. Dr. George I.M. Georgescu”, 700503 Iasi, Romania; 5 Department of Nephrology, “Carol Davila” University of Medicine and Pharmacy, 020021 Bucharest, Romania; gener.ismail@umfcd.ro; 6 Department of Nephrology, Fundeni Clinical Institute, 022328 Bucharest, Romania

**Keywords:** rituximab, hepatitis C, cryoglobulinemic vasculitis, remission, response rate

## Abstract

(1) Background. Hepatitis C infection often leads to extrahepatic manifestations, including cryoglobulinemic vasculitis. This systematic review aimed to assess the efficacy and safety of rituximab in treating hepatitis C-associated cryoglobulinemic vasculitis. (2) Methods. Following PRISMA guidelines, databases were searched for relevant studies. Eligibility criteria included studies on hepatitis C-associated cryoglobulinemic vasculitis treated with rituximab. (3) Results. Nine studies met the eligibility criteria and were included in this analysis. Rituximab was commonly administered at 375 mg/m^2^ weekly for one month. The results consistently demonstrated the efficacy of rituximab, whether as a standalone treatment or as part of a therapeutic regimen. The combination of rituximab with Peg-IFN-α and ribavirin significantly increased the complete response rate compared to Peg-IFN-α and ribavirin alone (54.5% vs. 33.3%, *p* < 0.05). The 3-year sustained response rate was notably higher in the rituximab combination group (83.3% vs. 40%). In another trial, rituximab achieved remission in 83.3% of patients at 6 months, compared to only 8.3% in the control group. The efficacy of rituximab was supported by long-term experience, with clinical benefits in patients with severe cryoglobulinemic vasculitis, including those resistant to standard therapies. Mild adverse events were generally reported, with rare severe reactions in some studies. (4) Conclusions: In conclusion, rituximab appeared to be effective and safe in managing hepatitis C-associated cryoglobulinemic vasculitis, either alone or with antiviral therapy.

## 1. Introduction

Hepatitis C infection affects more than 170 million individuals worldwide and it is a leading cause of chronic liver disease, cirrhosis, and hepatocellular carcinoma [1,2]. Contemporary treatment protocols involving direct-acting antiviral agents were linked to higher rates of sustained virologic response in patients with HCV infection and higher rates of clinical remission compared to previous antiviral regimens in those with HCV-associated cryoglobulinemic vasculitis [3,4].

HCV infection is associated with extrahepatic manifestations in up to 50% of patients leading to increased morbidity and mortality [5]. One of the most common extrahepatic manifestations of HCV infection is cryoglobulinemia. Types II and III cryoglobulinemia, also called mixed cryoglobulinemia, are the most frequent and are usually associated with hepatitis C infection [6]. The pathogenesis of immunological abnormalities associated with cryoglobulinemic vasculitis is not entirely understood [6]. Hepatitis C infection is associated with autoimmune phenomena. In particular, the hepatitis C virus lowers the B-cell activation threshold, by directly infecting lymphocytes and inducing self-reactivity through molecular mimicry. This mechanism can also be involved in the development of mixed cryoglobulinemia in hepatitis C infection [7].

Since the recognition of HCV infection, it has been increasingly established that “essential” cryoglobulinemia is primarily linked to HCV infection. In patients with mixed cryoglobulinemia, anti-HCV antibodies and HCV-RNA are detected in a substantial proportion of cases (90%) [8]. Additionally, HCV-RNA and anti-HCV antibodies are concentrated in cryoglobulins [9]. Some studies showed that B cells from most patients with HCV infection expressed elevated levels of the activation markers CD69, CD71, and CD86 and of the chemokine receptor CXCR3, when compared to healthy controls or with patients with hepatitis B infection. The same study reported that the HCV receptor CD81 activates human B cells even in the absence of the B cell antigen receptor colligation, suggesting that B cells are a target for HCV infection [10]. A monoclonal B cell proliferation is present in the peripheral blood lymphocytes and liver biopsy of patients infected with HCV. Additionally, one study observed that B cell proliferation was associated with a higher serum cryoglobulin level and cryoglobulinemia-related symptoms [10].

Circulating mixed cryoglobulins are detected in 40–60% of patients with chronic hepatitis-C infection [11]. Nevertheless, cryoglobulinemic vasculitis is only observed in 5–10% of such cases [11].

While cryoglobulinemia may remain asymptomatic, in symptomatic cases, the range of manifestations varies from mild symptoms, such as purpura or arthralgias, to more severe manifestations like glomerulonephritis or overt vasculitic disease [11]. The main manifestations of mixed cryoglobulinemia include purpura, arthralgias, and weakness [1]. Kidney damage is seen in 20–25% of cases with mixed cryoglobulinemia and presenting with various amounts of hematuria and proteinuria [11]. Membranoproliferative glomerulonephritis is the most frequently observed lesion on a kidney biopsy [12]. The pathogenesis of cryoglobulin-induced injury is not entirely understood. Immune complexes are deposited in the glomeruli, triggering an inflammatory response and activating the complement system [13,14]. Some experimental studies show that IgG3-dominant immune complexes trigger glomerulonephritis [15]. Progression to end-stage renal disease is seen after a decade or more [11].

Asymptomatic cryoglobulinemic vasculitis does not require specific therapy, whereas symptomatic cases need management according to disease severity and organ damage and immunosuppression may be needed [16,17].

Given the B cell proliferation associated with hepatitis C infection, Rituximab, an antiCD-20 monoclonal antibody found on the surface of B cells was been used as treatment for cryoglobulinemic vasculitis. Rituximab was used as treatment for both hepatitis C-associated cryoglobulinemic vasculitis and for essential mixed cryoglobulinemia [18]. Moreover, the KIDGO 2022 Clinical Guidelines for the Prevention, Diagnosis, Evaluation, and Treatment of Hepatitis C in Chronic Kidney Disease, Rituximab is recommended as a first-line therapy for patients with cryoglobulinemic vasculitis and glomerulonephritis (1C) [19].

However, there are numerous questions regarding the use of Rituximab in this particular set of patients. The role of rituximab as first-line therapy or as a therapy for relapses must be further defined. Existing studies have employed Rituximab in various ways for the management of cryoglobulinemic vasculitis. Some studies utilize Rituximab as induction therapy, while others use it to treat relapses or cases of disease that have not responded to corticosteroid treatment. However, there is a lack of data regarding the long-term effectiveness of treatment and the risk of recurrence in these patients.

The main aim of our study was to systematically evaluate the literature on the efficiency and safety of rituximab in cryoglobulinemic vasculitis, exclusively in patients with hepatitis C infection.

## 2. Materials and Methods

In the present systematic review, we adhered to the revised guidelines provided by the Preferred Reporting Items for Systematic Reviews and Meta-Analyses (PRISMA) [20]. This encompassed all aspects, spanning from the methodology of our search procedure to data collection and presentation. The protocol of the systematic review was registered in the PROSPERO register of systematic reviews (CRD42023459957).

### 2.1. Data Sources and Search Strategy

A comprehensive inquiry was undertaken within the time span extending from the 1 April 2023 to 31 July 2023, across MEDLINE (PubMed), Embase, Scopus, and Cochrane databases. Complementary to the databases above, potential eligible citations were sought through the screening of Google Scholar and ClinicalTrials.gov databases. Language filters were not applied in the search process. Also, citations within pivotal studies were analyzed to identify additional references that align with our pre-established eligibility criteria. To construct a comprehensive search strategy, a range of keyword combinations and controlled terminology were employed, including Medical Subject Headings (MeSH terms) for MEDLINE and Emtree terms for Embase, as follows: “rituximab”, “monoclonal antibodies”, “immunotherapy”, “biologic agents”, “cryoglobulinemia”, “mixed cryoglobulinemia”, “vasculitis”, “mixed essential cryoglobulinemia”, “mixed cryoglobulinemia”, “cryoglobulinemic vasculitis”, “hepatitis C”, “hepatitis C-induced vasculitis”, “hepatitis C-associated cryoglobulinemia”, “efficacy”, “response rate”, “remission”, “clinical improvement”, “outcomes”, “safety”, “adverse events”, and “side effects”. The complete search strategy across all mentioned databases was reported in Supplementary Table S1.

### 2.2. Eligibility Criteria and Outcomes

We predefined explicit inclusion and exclusion criteria before initiating the search and subsequent data extraction process. Two independent investigators used these criteria to determine whether the retrieved study met the eligibility requirements and were included in the present analysis. The following inclusion criteria were applied: (1) randomized or observational study design; (2) studies involving human participants with hepatitis C-associated cryoglobulinemic vasculitis; (3) studies investigating rituximab as a therapeutic intervention for the management of hepatitis C-associated cryoglobulinemic vasculitis (alone or as a component of a therapeutic protocol); (4) studies with relevant comparators, including other immunosuppressive drugs or placebo (when available); and (5) studies reporting at least one of the following outcomes: improvement in vasculitis symptoms, remission rate, and median duration of remission, relapse rate of cryoglobulinemic vasculitis, reduction in cryoglobulin levels, as well as studies reporting side effects linked to rituximab therapy. Also, some critical exclusion criteria were applied in selecting studies for analysis: (1) studies not investigating the use of rituximab for the management of hepatitis C-associated cryoglobulinemic vasculitis; (2) studies not reporting relevant clinical outcomes; (3) abstracts, editorials, conference proceedings, and posters not available in full text; (4) unpublished data or missing outcome data.

### 2.3. Data Collection and Synthesis

The following data were extracted from qualified research papers that fulfilled the inclusion criteria: primary investigator, year of publication, population sample size and their age, clinical context, therapeutic regimen used to treat cryoglobulinemic vasculitis (including rituximab dose when reported), duration of follow-up and investigated outcomes. The methodological aspects of included studies that could affect the outcomes were extracted and analyzed. Collected data were presented as median or mean values, percentages, odds ratio (OR) or risk ratio (RR), and *p*-values (when available).

### 2.4. Quality Assessment

The quality assessment of the included studies was conducted based on their designs. In the case of randomized clinical trials, the risk of bias was evaluated using the revised Cochrane risk-of-bias tool for randomized trials (RoB 2) [21]. Conversely, for observational, non-randomized studies, the overall quality of the research was appraised using the Newcastle–Ottawa scale (NOS). This scale comprises critical criteria grouped into three main domains: selecting participants, comparing groups, and investigating outcomes [22].

## 3. Results

**Figure 1:**
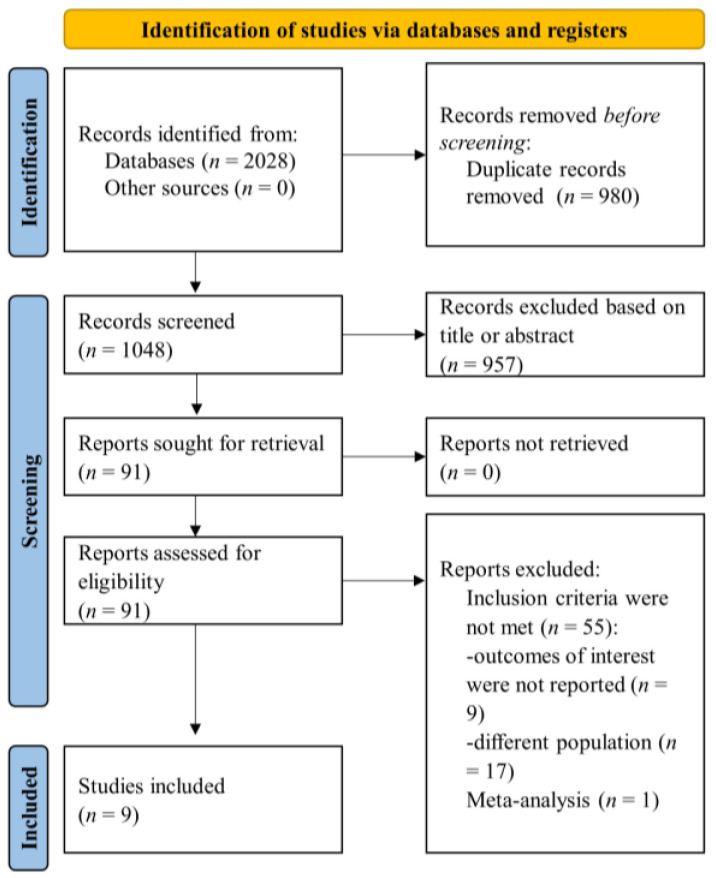
Flow diagram of selected studies in the present analysis.

A thorough and systematic search was conducted across designated databases, yielding an initial pool of 2028 records. Subsequently, duplicate publications were eliminated, resulting in a filtered dataset comprising 464 references. These articles underwent a second assessment in line with predefined inclusion and exclusion criteria, based on a two-step approach. In the first step, two independent investigators evaluated titles and abstracts for eligibility criteria. References which fulfilled the inclusion criteria were examined in full-text in the second step. Subsequent to the full-text screening, nine studies [23,24,25,26,27,28,29,30,31] met the eligibility criteria and were included in the present analysis, as displayed in Figure 1 of the search flowchart.

The features of included studies, encompassing the publication year, study design, clinical context of enrolled participants and their age, therapeutic regimen used, rituximab dose and follow-up duration were outlined in Table 1. In addition, Table 2 provides an overview of the main findings reported in analyzed studies and the specific outcomes investigated.

The majority of studies had an observational design [24,25,27,28,30,31], while two studies were randomized [23,29], and one study was a secondary analysis of a randomized controlled trial with long-term results [26]. Concerning the therapeutic regimen used, rituximab was administered in combination with pegylated interferon-α (Peg-IFN-α) and/or antiviral therapy in two studies [23,27], whereas in one study, rituximab was administered after therapy with methylprednisolone, paracetamol, and chlorpheniramine [31]. The clinical profile of patients in these studies predominantly comprised participants with severe hepatitis C-related cryoglobulinemic vasculitis, experiencing relapses despite standard therapy, or resistant to standard treatment. Regarding the rituximab dose, most authors employed a regimen of 375 mg/m^2^ administered once a week for one month [25,27,28,29,30], while one study employed a dosage of 250 mg/m^2^ given twice, one week apart [31].

Data from the available randomized clinical trials consistently demonstrated the efficacy of rituximab therapy, whether used as a standalone treatment or as part of a therapeutic regimen [23,29]. When comparing the treatment outcomes, the addition of rituximab to the Peg-IFN-α and ribavirin combination (PIRR regimen) demonstrated a significantly higher complete response rate (54.5%) than that observed in patients solely treated with Peg-IFN-α and ribavirin (33.3%), *p* < 0.05 [23]. In addition, the partial response rate in the PIRR group was 22.7%, in contrast to 33.3% observed in patients treated with Peg-IFN-α and ribavirin. This difference can be attributed to the higher complete response rate in the PIRR arm. Of particular significance, the 3-year sustained response rate in patients that received PIRR regimen was more than two-fold higher when compared to those who underwent antiviral therapy alone with Peg-IFN-α and ribavirin therapy (83.3% vs. 40%). Despite the advantages of combined therapy with rituximab, it is noteworthy that five patients from each treatment group did not respond to the therapy, and they exhibited similar clinical and biological parameters before and after the treatment [23]. Rituximab demonstrated a good tolerability, with only three mild adverse events reported after the first infusion, and two patients experiencing fever following the third and fourth infusions [23].

In another randomized controlled trial, 83.3% patients treated with rituximab were in remission at 6 months, compared to only 8.3% in the control group (best available therapy), *p* < 0.001 [29]. Also, the Birmingham Vasculitis Activity Score (BVAS) exhibited a significant decline in the rituximab arm, in contrast to patients from the control group (*p* < 0.02). Remission was maintained for a median duration of 7 months (IQR, 4.5–10), and relapses were effectively managed with the supplementary rituximab course [29].

Data from a long-term experience center suggest a benefit of combined therapy with antiviral therapy following a rituximab course in patients with severe cryoglobulinemic vasculitis [24]. In these patients (that had a mean baseline of BVAS 19.0 ± 5.76), antiviral therapy after a rituximab course, induced clinical remission in 83.3% cases. Moreover, compared to glucocorticoids, complete clinical response was achieved in 73.3% patients who received rituximab (vs. 13.0% in glucocorticoids group) [24].

For patients who do not respond to the initial standard therapy or experience relapses, the addition of rituximab to their treatment regimen could offer substantial benefits. Rituximab induced a long-term clinical response in 43.3% patients, extending up to 62.4 months. Additionally, the efficacy of second and third rituximab retreatments was observed in two-thirds of the patients [26]. In another study investigating resistant patients or with relapse to previous standard therapy, rituximab combined with Peg-IFN-α and ribavirin induced complete clinical response in 62.5% cases, with two clinical relapses during follow-up [27]. Serum cryoglobulins were undetectable in 62.5% patients, while a decrease of at least 50% was reported in 31.2% cases [27]. In patients resistant to IFN-α therapy, treatment with rituximab achieved complete remission and cryocrit decrease in 80% patients, with sustained remission during follow-up in 75% cases [28]. Low-dose rituximab (250 mg/m^2^ given twice, one week apart) was also efficient in refractory cryoglobulinemic vasculitis, with complete or partial clinical remission in 85% patients [31].

Rituximab had a good safety profile in majority of studies, with mild adverse reactions and fever as the most frequently adverse events reported [23,25,27,28]. Nevertheless, some studies reported severe fever following the second rituximab infusion [29], serum sickness syndrome, severe flare of vasculitis [30], anaphylaxis, neutropenia, thrombophlebitis, pneumonia, and flu-like syndrome [31].

**Figure 2:**
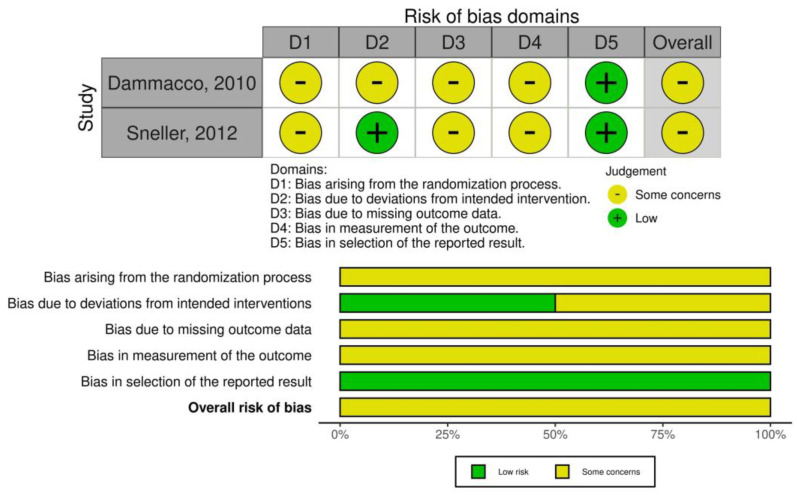
Risk of bias in randomized trials (using RoB 2 tool). Dammacco, 2010 [23]; Sneller, 2012 [29].

Regarding the risk of bias in randomized clinical trials, there were some concerns as appraised using the RoB 2 tool (Figure 2). Likewise, observational studies had a fair to low quality evaluated by NOS designed for non-randomized trials (Table S2).

## 4. Discussion

Treatment of HCV-associated cryoglobulinemic vasculitis usually consists of virus-directed and a B-cell clone-directed regimens. As such, the main goal of the treatment is the achievement of clinical and immunological remission, following a sustained virologic response obtained with antiviral therapy. Our study showed that treatment with Rituximab can be efficient in patients with HCV-associated cryoglobulinemic vasculitis, either as induction therapy or as therapy for relapses or resistant forms. Moreover, Rituximab is effective either alone or in combination with Peg-IFNα and ribavirin. Rituximab has demonstrated the effectiveness in inducing a clinical response in manifestations of cryoglobulinemic vasculitis. Rituximab therapy has proven to be effective in reducing the manifestations of skin lesions, polyneuropathy, and promoting the remission of kidney injury. The safety profile of Rituximab across various studies has been favorable, with only a few reported adverse effects.

When considering immunosuppression in the context of the hepatitis C virus, it is essential to take into account the fact that the virus has a tropism for lymphocytes, potentially leading to hepatitis C-related lymphomagenesis [32]. In these cases, the choice of treatment should be individualized. For instance, when the disease follows an indolent course, initiating treatment with direct-acting antivirals (DAAs) is advisable. However, if the lymphoproliferative disease has progressed to an irreversible point, consideration should be given to immune-chemotherapy [33].

The introduction of direct-acting antivirals (DAAs) has brought about a significant transformation in the landscape of chronic hepatitis C infection. The use of DAAs results in a sustained virologic response in over 90% of cases. However, when symptomatic mixed cryoglobulinemia is present, DAA therapy may not always be sufficient. Some studies indicate that there is no substantial improvement in mixed cryoglobulinemic vasculitis, nephritis, and peripheral neuropathy, even after achieving a sustained virologic response through DAA treatment [34]. Essentially, cryoglobulinemia may persist or reappear despite DAA treatment.

In cases where DAAs alone are insufficient to manage cryoglobulinemic symptoms, immunosuppressive therapy becomes necessary. Treatment options encompass glucocorticoids, anti-CD20 monoclonal antibodies, cyclophosphamide, and in severe, life-threatening cases, the consideration of apheresis [33].

Around 25–30% of patients with HCV infection test positive for non-organ-specific autoantibodies, including anti-nuclear antibodies (ANA), smooth muscle antibodies (SMA), and liver–kidney microsomal antibody type 1 (LKM1). An intriguing observation is the similarity between the antibodies present in some HCV cases and those seen in autoimmune hepatitis [35]. Moreover, there is a heightened activation of the immune system in HCV patients who are positive for anti-LKM1 antibodies. In this subgroup of patients, antiviral treatment proves beneficial. In cases of necroinflammatory flares, these can be managed with corticosteroid therapy, after which antiviral treatment can be resumed [36]. For patients with associated non-organ-specific autoantibodies, combined antiviral treatment with IFN-ribavirin is not only effective but also demonstrates a good safety profile. However, it is worth noting that the long-term response is less favorable in patients infected with HCV-1 [37].

Immunosuppression is known to worsen hepatitis C, especially when B-cells are depleted using rituximab therapy. However, as reported in Dammacco et al., no clinical viral reactivation was observed when a combination therapy including rituximab was administered [23]. In the studies included in our systematic review, there was no evidence of viral reactivation and rituximab appears to be safe in patients with HCV infection, although there is a risk for transient elevation in liver enzymes. Regarding the safety profile, rituximab was well tolerated, with a few adverse effects throughout the analyzed studies, especially when compared to other regimens. In Dammacco et al., three patients experienced mild adverse events after the infusion of rituximab, but patients completed the study until the end of follow-up [23]. In Senne et al., adverse events were reported in 27.3% of patients (two patients with serum sickness syndrome and four patients with a severe flare of mixed cryoglobulinemia) [30]. Nevertheless, adverse events were more frequent in the high-dose Rituximab protocol [30].

Concerning Rituximab dosage, Visentini et al., proposed a low-dose rituximab regimen using 250 mg/m^2^ given twice (one week apart), and the results showed a complete response in 81% cases [31]. However, to the best of our knowledge, there are no randomized controlled trials at this point comparing low-dose versus high-dose rituximab in patients with hepatitis C-associated cryoglobulinemic vasculitis.

A randomized controlled trial by De Vita et al., (6) included 59 patients with cryoglobulinemic vasculitis and compared rituximab therapy with conventional therapy (that included glucocorticoids, azathioprine, cyclophosphamide or plasmapheresis). The primary outcome was the survival of treatment at 12 months and was statistically higher in the rituximab group (64.3% vs. 3.5%). Rituximab proved also effective in patients with failed initial standard therapy. Although 93% patients enrolled in this study were positive for hepatitis C, we did not include it in our systematic review, because of the non-hepatitis C patients (without a subgroup analysis) [38]. The findings in our systematic review are in line with other studies on this topic, showing the remission of vasculitis phenomena when combining antiviral therapy with Rituximab [39,40].

Other studies enrolled hepatitis C positive patients with associated cryoglobulinemic glomerulonephritis. These studies were excluded from our analysis because not all of their patients had vasculitis. Nonetheless, it is reasonable to expect that certain outcomes may still be relevant to cryoglobulinemic vasculitis associated with HCV infection. Quartuccio et al., included five patients with biopsy-proven glomerulonephritis in HCV-related type II mixed cryoglobulinemia that were treated with rituximab (without corticosteroids when possible) [41]. A rapid and sustained renal response was observed in all patients after 6 months. Also, upon follow-up renal biopsies, notable histological improvements were reported [41]. Roccatello et al., included five patients with HCV infection and cryoglobulinemic glomerulonephritis that were treated with Rituximab. Results showed a significant decrease in the proteinuria levels, sedimentation rate, and cryocrit levels at 2, 6, and 12 months [42].

It is worth noting that the studies included in our systematic review did not involve antiviral therapy using DAA. Patients with HCV and cryoglobulinemic vasculitis typically achieve a high sustained virological response through DAA treatment. However, even with successful DAA treatment, certain factors such as circulating cryoglobulins, rheumatoid factor activity, and clonal B-cell proliferation may persist, leading to clinical relapses of cryoglobulinemic vasculitis. In these cases, Rituximab can be a suitable treatment option. However, there is uncertainty about the timing of Rituximab in relation to DAA treatment [43].

In a prospective study by Saadoun et al. [43], 4 out of 24 patients received Rituximab in addition to DAA treatment and achieved a sustained virological response, along with reduced cryocrit levels and clinical improvement. Similarly, in a study by Sise et al. [44], four out of 12 patients underwent DAA + Rituximab therapy and observed similar positive outcomes as in the previous study. However, as far as our knowledge goes, there are no studies directly comparing the DAA treatment plus Rituximab to DAA treatment alone for cryoglobulinemic vasculitis. The existing literature on this topic primarily consists of case reports and case series.

Our study has several limitations. First, most studies included in our analysis had an observational design, with only two randomized controlled trials included and one retrospective analysis of a randomized controlled trial. Second, throughout the studies, rituximab was either used as an induction therapy or as a therapy for relapses or resistant forms of HCV-associated cryoglobulinemic vasculitis. Hence, direct comparisons between these regimens are not feasible, and further research is imperative to ascertain whether rituximab exhibits a comparable efficacy profile when employed as an induction treatment, in managing relapses, or addressing resistant forms. Third, two studies included in our systematic review did not report the rituximab dose used. Another limitation arises from the variability in regimens, diverse outcome measures, and the inclusion of a relatively small number of patients in each study. Consequently, it was not feasible to carry out a meta-analysis.

## 5. Conclusions

In conclusion, our systematic review underscores the efficacy and safety of rituximab in managing HCV-associated cryoglobulinemic vasculitis. This monoclonal antibody therapy, either alone or in combination with antiviral treatment, consistently demonstrated a positive impact on disease remission and clinical outcomes, offering a therapeutic alternative to patients facing relapses or resistant forms of cryoglobulinemic vasculitis. Although some studies indicated the potential benefits of lower-dose rituximab regimens, further investigations are warranted to elucidate the optimal dosage and therapeutic strategies. Despite the predominance of observational studies and heterogeneity in treatment regimens, our findings provide valuable insights into the potential of rituximab as a valuable therapeutic option for this challenging condition, emphasizing the need for more rigorous research, particularly randomized controlled trials, to solidify its place in the clinical management of hepatitis C-associated cryoglobulinemic vasculitis. Further on, it would be beneficial for future research to focus on comparing Rituximab therapy plus DAA versus DAA therapy alone in patients with cryoglobulinemic vasculitis.

## Figures and Tables

**Table 1:** General characteristics of included studies in the systematic review.

Author, Year	Design	Patients, No	Age, Median/Mean ± SD	Clinical Context	Regimen Used	Rituximab Dose	Follow-Up
Dammacco et al., 2010 [23]	Randomized, single-center trial	37	63 (PIRR)	Induction therapy	PIRR (*n* = 22)	375 mg/m^2^ once a week for 1 month, then two infusions 5-times-per-month	3 years
			59 (Peg-IFN-α/RBV)				
Ignatova et al., 2017 [24]	Observational, single-center	72	49.4 ± 10.3	Patients with HCV-associated CV	Rituximab vs. traditional immunosuppressive therapy or antiviral therapy	NR	2.8 ± 3.6 years
Petrarca et al., 2010 [25]	Observational, prospective, single-center	19	63	Induction therapy	Rituximab	375 mg/m^2^ once a week for 1 month	6–48 months
Quartuccio et al., 2015 [26]	Retrospective analysis of a randomized controlled trial (long-term results)	30	63 ± 11	Relapse of severe HCV-related CV	Rituximab	NR	72.6 months
Saadoun et al., 2008 [27]	Observational	16	58 ± 10.5	Resistant patients or with relapse to previous standard therapy	Rituximab combined with Peg-IFN-α and RBV	375 mg/m^2^ weekly for 4 weeks	19.4 months
Sansonno et al., 2003 [28]	Observational, prospective	20	NR	Patients resistant to IFN-α therapy	Rituximab	375 mg/m^2^ weekly for 4 weeks	12 months
Sneller et al., 2012 [29]	Open-label, single-center, randomized trial	24	53 (rituximab group)	Prior antiviral therapy failed	Rituximab (*n* = 12) vs. best available therapy (*n* = 12)	375 mg/m^2^ on days 1, 8, 15, and 22	6 months
			51 (control group)				
Sene et al., 2009 [30]	Observational, prospective	22	56.7 ± 11.85	HCV patients with biopsy-proven MC vasculitis	Rituximab	375 mg/m^2^ weekly for 4 weeks	NR
Visentini et al., 2015 [31]	Observational, multicenter	52	69 ± 8.4	Refractory MC vasculitis in HCV patients	Rituximab (after treatment with methylprednisolone, paracetamol and chlorpheniramine)	250 mg/m^2^ given twice (one week apart)	12 months

CV = cryoglobulinemic vasculitis; HCV = hepatitis C virus; IFN-α = interferon-α; MC = mixed cryoglobulinemia; Peg-IFN-α = pegylated interferon-α; PIRR = combination of Peg-IFN-α and RBV with rituximab; RBV = ribavirin.

**Table 2:** Results reported in analyzed studies from the present systematic review.

Author, Year	Regimen Used	Results	
Dammacco, 2010 [23]	PIRR vs. Peg-IFN-α/RBV	Complete response was reported in 54.5% patients from PIRR group, as compared to 33.3% from Peg-IFN-α/RBV therapy group (following 1 year).	*p* < 0.05
		Response maintenance rate: 83.3% in PIRR group vs. 40% in Peg-IFN-α/RBV group (at 36 months)	*p* < 0.01
		Partial response rate: 22.7% in PIRR group vs. 33.3% in Peg-IFN-α/RBV group	
		Safety: rituximab had a good safety profile, with mild adverse reactions (*n* = 3) following the first infusion (and fever in two patients following the third and fourth infusions).	
Ignatova, 2017 [24]	Rituximab vs. glucocorticoids	Complete clinical response in 73.3% patients receiving rituximab as compared to 13.0% patients treated with glucocorticoids.	
		Polyneuropathy remission: 50% in rituximab group vs. 0% in control group	*p* = 0.021
		Kidney injury remission: 81.8% in rituximab group vs. 32.1% in control group	*p* = 0.02
		Severe skin lesions remission: 80% in rituximab group vs. 50% in control group.	*p* = 0.19
	AVT + rituximab	In patients with BVAS 19.0 ± 5.76, AVT following rituximab therapy induced clinical remission in 83.3% patients	
		AVT in combination with rituximab was more efficient in severe cases of vasculitis.	
Petrarca, 2010 [25]	Rituximab	At the end of follow-up, complete remission was reported in 63.1% patients (*n* = 12), while partial response was reported in 36.9% patients (*n* = 7).	
		From 5 patients with renal injury, 2 patients displayed a complete response, while a partial response was reported in 3 patients.	
		Nine patients had a complete cryocrit response, whereas in two patients cryocrit decreased with at least 50%.	
		Safety: rituximab therapy was not associated with notable side effects.	
Quartuccio, 2015 [26]	Rituximab	A long-term response to rituximab was reported in 13 patients (43.3%) during a mean follow-up period of 62.4 months (without clinical relapse).	
		Retreatment with rituximab was required in 17 patients: single retreatment (10 patients), two retreatments (6 patients), and three retreatments (1 patient).	
		From patients requiring retreatment, rituximab was efficient in 2/3 of patients (including complete remission in 1/3 of cases).	
		Safety: clinically relevant adverse events: urinary or respiratory tract infections (10% of patients) and chronic hypogammaglobulinemia (7% of patients)	
Saadoun, 2008 [27]	Rituximab combined with Peg-IFN-α and RBV	Complete clinical response was reported in 10 patients (62.5%), partial response—in 5 patients (31.2%), while 1 patient was non-responsive.	
		Symptoms and signs improved: leg ulcers in all patients; purpura in 84.6% cases; renal injury in 57.2% patients; polyneuropathy in 38.4% cases; hematuria in all patients; proteinuria in 71.4% cases.	
		Serum cryoglobulins were undetectable in 62.5% patients, while 31.2% patients experienced a decrease of at least 50% from baseline.	
		Clinical relapse was observed in 2 patients during follow-up.	
		Safety: reported adverse events were linked to Peg-IFN-α therapy, including peripheral neuropathy (*n* = 1) and worsening of skin psoriasis (*n* = 1).	
Sansonno, 2003 [28]	Rituximab	Complete response and cryocrit decrease were reported in 16 patients (80%).	
		Symptoms and signs improved: purpura in 87.5% cases; peripheral neuropathy in 50% cases.	
		Sustained remission during follow-up was documented in 12 patients (75%).	
		Safety: no severe adverse events were reported in patients receiving rituximab.	
Sneller, 2012 [29]	Rituximab vs. best available therapy	In the rituximab group, 10 patients (83.3%) were in remission at 6 months, as compared to 1 patient (8.3%) in the control group.	*p* < 0.001
		Remission was sustained for a median duration of 7 months (relapses were treated with supplementary rituximab course).	
		BVAS decreased in the rituximab group, as compared to control group.	*p* < 0.02
		Safety (rituximab group): one patient had severe fever following the second rituximab infusion; HCV RNA levels were similar in rituximab group.	
Sene, 2009 [30]	Rituximab	Safety: systemic side effects were documented in 6 patients (27.3%), 2 patients had serum sickness syndrome and 4 patients had a severe flare of MC vasculitis (more frequent in high-dose rituximab protocol).	
Visentini, 2015 [31]	Rituximab (after methylprednisolone, paracetamol and chlorpheniramine)	Clinical response (complete or partial) was reported in 85% patients (complete response in 81% cases).	
		At 1-year follow-up, 17 patients were in remission, while relapse was observed in 17 cases.	
		Cryoglobulin levels were undetectable in 44% patients, and in 10% cases a decrease of at least 50% was reported.	
		Safety: seven patients developed side effects, including anaphylaxis, serum sickness syndrome, neutropenia, thrombophlebitis, pneumonia, and flu-like syndrome.	

AVT = antiviral therapy; BVAS = Birmingham Vasculitis Activity Score; HCV = hepatitis C virus; IFN-α = interferon-α; MC = mixed cryoglobulinemia; Peg-IFN-α = pegylated interferon-α; PIRR = combination of Peg-IFN-α and RBV with rituximab; RBV = ribavirin.

## Data Availability

Not applicable.

## Supplementary Materials

The following supporting information can be downloaded at: https://www.mdpi.com/article/10.3390/jcm12216806/s1. Table S1: Databases and search strategy; Table S2: Quality assessment [24,25,26,27,28,30,31].
