# Supplementary material for: Therapeutic Potential of Rituximab in Managing Hepatitis C-Associated Cryoglobulinemic Vasculitis: A Systematic Review
Source: J Clin Med. 2023 Oct 27;12(21):6806. doi: 10.3390/jcm12216806 (PMC10648453; doi:10.3390/jcm12216806)
Supplement: Supplementary file 1 [file jcm-12-06806-s001.zip › Table S1 Databases and search strategy.pdf]

Table S1. Databases and search strategies used in present systematic review.

Database	Coverage	Search run	Records
**MEDLINE**	1946 to present	("Rituximab"[Mesh]OR monoclonal antibody OR immunotherapy OR biologic agents) AND ("Cryoglobulinemia"[Mesh] OR mixed cryoglobulinemia OR mixed essential cryoglobulinemia OR cryoglobulinemic vasculitis) AND (hepatitis C OR hepatitis C-induced vasculitis OR hepatitis C-associated cryoglobulinemia) AND (efficacy OR safety OR response rate OR remission OR clinical improvement OR outcomes OR adverse events OR side effects)	317
		("Rituximab"[Mesh] OR monoclonal antibody OR immunotherapy OR biologic agents) AND ("Cryoglobulinemia"[Mesh] OR mixed cryoglobulinemia OR mixed essential cryoglobulinemia OR cryoglobulinemic vasculitis) AND (hepatitis C OR hepatitis C-induced vasculitis OR hepatitis C-associated cryoglobulinemia)	825
		("Rituximab"[Mesh]) AND ("Cryoglobulinemia"[Mesh] OR cryoglobulinemic vasculitis) AND (hepatitis C OR hepatitis C-induced vasculitis OR hepatitis C-associated cryoglobulinemia)	123
			Total records = 1265
**Embase**	1966 to present	('rituximab'/exp OR monoclonal antibody OR immunotherapy OR biologic agents) AND ('cryoglobulinemia'/exp OR mixed cryoglobulinemia OR mixed essential cryoglobulinemia OR cryoglobulinemic vasculitis) AND (hepatitis C OR hepatitis C-induced vasculitis OR hepatitis C-associated cryoglobulinemia) AND (efficacy OR safety OR response rate OR remission OR clinical improvement OR outcomes OR adverse events OR side effects)	1
		('rituximab'/exp OR monoclonal antibody OR immunotherapy OR biologic agents) AND ('cryoglobulinemia'/exp OR mixed cryoglobulinemia OR mixed essential cryoglobulinemia OR cryoglobulinemic vasculitis) AND (hepatitis C OR hepatitis C-induced vasculitis OR hepatitis C-associated cryoglobulinemia)	2
		(Rituximab) AND (Cryoglobulinemia OR cryoglobulinemic vasculitis) AND (hepatitis C OR hepatitis C-induced vasculitis OR hepatitis C-associated cryoglobulinemia)	725
			Total records = 728
**Cochrane library**	1967 to present	(Rituximab OR monoclonal antibody OR immunotherapy OR biologic agents) AND (Cryoglobulinemia OR mixed cryoglobulinemia OR mixed essential cryoglobulinemia OR cryoglobulinemic vasculitis) AND (hepatitis C OR hepatitis C-induced vasculitis OR hepatitis C-associated cryoglobulinemia) AND (efficacy OR safety OR response rate OR remission OR clinical improvement OR outcomes OR adverse events OR side effects)	12
		(Rituximab OR monoclonal antibody OR immunotherapy OR biologic agents) AND (Cryoglobulinemia OR mixed cryoglobulinemia OR mixed essential cryoglobulinemia OR cryoglobulinemic vasculitis) AND (hepatitis C OR hepatitis C-induced vasculitis OR hepatitis C-associated cryoglobulinemia)	13

		(Rituximab) AND (Cryoglobulinemia OR cryoglobulinemic vasculitis) AND (hepatitis C OR hepatitis C-induced vasculitis OR hepatitis C-associated cryoglobulinemia)	10
			Total records = 35
**Scopus**	From the inception till present	(Rituximab OR monoclonal antibody OR immunotherapy OR biologic agents) AND (Cryoglobulinemia OR mixed cryoglobulinemia OR mixed essential cryoglobulinemia OR cryoglobulinemic vasculitis) AND (hepatitis C OR hepatitis C-induced vasculitis OR hepatitis C-associated cryoglobulinemia) AND (efficacy OR safety OR response rate OR remission OR clinical improvement OR outcomes OR adverse events OR side effects)	0
		(Rituximab OR monoclonal antibody OR immunotherapy OR biologic agents) AND (Cryoglobulinemia OR mixed cryoglobulinemia OR mixed essential cryoglobulinemia OR cryoglobulinemic vasculitis) AND (hepatitis C OR hepatitis C-induced vasculitis OR hepatitis C-associated cryoglobulinemia)	0
		(Rituximab) AND (Cryoglobulinemia OR cryoglobulinemic vasculitis) AND (hepatitis C OR hepatitis C-induced vasculitis OR hepatitis C-associated cryoglobulinemia)	0
			Total records = 0
All databases: 2028 records			
