# Supplementary material for: Therapeutic Potential of Rituximab in Managing Hepatitis C-Associated Cryoglobulinemic Vasculitis: A Systematic Review
Source: J Clin Med. 2023 Oct 27;12(21):6806. doi: 10.3390/jcm12216806 (PMC10648453; doi:10.3390/jcm12216806)
Supplement: Supplementary file 1 [file jcm-12-06806-s001.zip › Table S2. Quality assessment.pdf]

Table S2. Quality assessment of cohort studies using Newcastle-Ottawa scale.

Study	Representativeness of the exposed cohort	Selection of the non-exposed cohort	Ascertainment of exposure	Presence of outcome of interest at the start	Comparability of cohorts	Assessment of outcome	Follow-up long enough for outcomes to occur	Adequacy of follow-up	Total
Ignatova, 2017 [24]	*	*	*	*	*		*		6
Petrarca, 2010 [25]	*	NA	*	*	NA	*	*	*	6
Quartuccio, 2015 [26]	*	NA	*	*	NA	*	*	*	6
Saadoun, 2008 [27]	*	NA	*	*	NA	*	*	*	6
Sansonno, 2003 [28]	*	NA	*	*	NA		*	*	5
Sene, 2009 [30]	*	NA	*	*	NA	*		*	5
Visentini, 2015 [31]	*	NA	*	*	NA	*	*	*	6

Good quality: 3 or 4 stars in selection domain AND 1 or 2 stars in comparability domain AND 2 or 3 stars in outcome/exposure domain. Fair quality: 2 stars in selection domain AND 1 or 2 stars in comparability domain AND 2 or 3 stars in outcome/exposure domain. Poor quality: 0 or 1 star in selection domain OR 0 stars in comparability domain OR 0 or 1 stars in outcome/exposure domain.
